# Economic Evaluations of Digital Health Interventions for Patients With Heart Failure: Systematic Review

**DOI:** 10.2196/53500

**Published:** 2024-04-30

**Authors:** Neily Zakiyah, Dita Marulin, Mohammed Alfaqeeh, Irma Melyani Puspitasari, Keri Lestari, Ka Keat Lim, Julia Fox-Rushby

**Affiliations:** 1 Department of Pharmacology and Clinical Pharmacy Faculty of Pharmacy Universitas Padjadjaran Bandung Indonesia; 2 Center of Excellence for Pharmaceutical Care Innovation Universitas Padjadjaran Bandung Indonesia; 3 Department of Population Health Sciences Faculty of Life Sciences and Medicine King's College London London United Kingdom

**Keywords:** digital health, telemonitoring, telehealth, heart failure, cost-effectiveness, systematic review, mobile phone

## Abstract

**Background:**

Digital health interventions (DHIs) have shown promising results in enhancing the management of heart failure (HF). Although health care interventions are increasingly being delivered digitally, with growing evidence on the potential cost-effectiveness of adopting them, there has been little effort to collate and synthesize the findings.

**Objective:**

This study’s objective was to systematically review the economic evaluations that assess the adoption of DHIs in the management and treatment of HF.

**Methods:**

A systematic review was conducted using 3 electronic databases: PubMed, EBSCOhost, and Scopus. Articles reporting full economic evaluations of DHIs for patients with HF published up to July 2023 were eligible for inclusion. Study characteristics, design (both trial based and model based), input parameters, and main results were extracted from full-text articles. Data synthesis was conducted based on the technologies used for delivering DHIs in the management of patients with HF, and the findings were analyzed narratively. The PRISMA (Preferred Reporting Items for Systematic Reviews and Meta-Analyses) guidelines were followed for this systematic review. The reporting quality of the included studies was evaluated using the CHEERS (Consolidated Health Economic Evaluation Reporting Standards) guidelines.

**Results:**

Overall, 27 economic evaluations were included in the review. The economic evaluations were based on models (13/27, 48%), trials (13/27, 48%), or a combination approach (1/27, 4%). The devices evaluated included noninvasive remote monitoring devices (eg, home telemonitoring using digital tablets or specific medical devices that enable transmission of physiological data), telephone support, mobile apps and wearables, remote monitoring follow-up in patients with implantable medical devices, and videoconferencing systems. Most of the studies (24/27, 89%) used cost-utility analysis. The majority of the studies (25/27, 93%) were conducted in high-income countries, particularly European countries (16/27, 59%) such as the United Kingdom and the Netherlands. Mobile apps and wearables, remote monitoring follow-up in patients with implantable medical devices, and videoconferencing systems yielded cost-effective results or even emerged as dominant strategies. However, conflicting results were observed, particularly in noninvasive remote monitoring devices and telephone support. In 15% (4/27) of the studies, these DHIs were found to be less costly and more effective than the comparators (ie, dominant), while 33% (9/27) reported them to be more costly but more effective with incremental cost-effectiveness ratios below the respective willingness-to-pay thresholds (ie, cost-effective). Furthermore, in 11% (3/27) of the studies, noninvasive remote monitoring devices and telephone support were either above the willingness-to-pay thresholds or more costly than, yet as effective as, the comparators (ie, not cost-effective). In terms of reporting quality, the studies were classified as *good* (20/27, 74%), *moderate* (6/27, 22%), or *excellent* (1/27, 4%).

**Conclusions:**

Despite the conflicting results, the main findings indicated that, overall, DHIs were more cost-effective than non-DHI alternatives.

**Trial Registration:**

PROSPERO CRD42023388241; https://tinyurl.com/2p9axpmc

## Introduction

### Background

Heart failure (HF) is a complex and potentially fatal condition affecting approximately 26 million people worldwide and is associated with substantial morbidity and mortality [1,2]. The global impact of HF also imposes a significant economic burden, affecting patients and their families as well as communities [1,2]. Data from low- and middle-income countries (LMICs) indicate that mortality rates in patients with HF are higher in LMICs than in high-income countries (HICs) [3]. The overall estimated 1-year mortality rate for patients with HF in LMICs is 16.5% [3] compared to 8.3% in HICs. People living with HF also experience a significant decline in health-related quality of life (HRQoL) [4,5].

The growing availability of life-saving and evidence-based treatments, along with increasing life expectancy, suggests that there will be an increase in the prevalence of HF over time. This is attributed to the improved survival rates after an HF diagnosis and the aging population [1,6,7]. The rise in HF prevalence is leading to an increase in annual health care costs. In 2012, the estimated global annual cost of HF reached US $108 billion, with direct costs estimated at US $65 billion and indirect costs estimated at US $43 billion [8]. Considering a projected 22% increase in the cost of cardiovascular diseases (CVDs), HF-related expenses alone could potentially reach US $132 billion by 2030 [9]. Despite significant improvements in outcomes with medical therapy [7], readmission rates for patients hospitalized for HF are still high (ie, 50% within 6 months of discharge) [10,11]. Hospitalization rates have been shown to be correlated with disease severity, mortality, and lower HRQoL [5].

Considering the prevalence of HF and its substantial financial burden, there has been a global focus on cost-effective health care interventions aimed at providing effective and efficient support to patients, as well as a growing focus on the application of digital health interventions (DHIs), driven by the advanced integration of IT and mobile internet in health care practices [12]. The broad scope of digital health includes telehealth, teleconsultation, and telemonitoring using smartphone apps; telephone support; videoconferencing; noninvasive remote monitoring devices; wearables; implantable devices; and sensors [13,14]. In addition, emerging fields such as advanced computing sciences in big data, genomics for personalized medicine, and artificial intelligence have been recognized as DHIs [13,15-17]. DHIs are used by providers and other stakeholders to enhance access, reduce inefficiencies and costs, improve quality, and potentially incorporate personalized medicine to improve patients’ clinical outcomes [14].

By using DHIs in the management of HF, it may be possible to prevent the progression of a patient’s condition and potentially reduce health care costs [18]. HF is a chronic condition in which people often experience episodic deterioration. Improvement in disease monitoring can enable prompt identification of patient deterioration and facilitate timely interventions to restabilize the syndrome [19,20]. Implementing DHIs such as teleconsultation and remote monitoring can reduce unnecessary hospital visits, provide continuous disease monitoring, develop effective disease management, and improve clinical outcomes. However, because the landscape of DHIs is evolving rapidly, regulators, reimbursement authorities, and health care professionals often face challenges in evaluating the value of these technologies, as reflected in current recommendations in the international guidelines of the European Society of Cardiology and the American College of Cardiology [19,20]. Skepticism regarding the value of DHIs is partly driven by the limited large-scale studies that demonstrate a consistent impact and effectiveness [18].

Despite the growth in, and the integration of, DHIs in recent years, evidence from economic evaluations is limited. One systematic review found that telemedicine improved clinical outcomes and resulted in cost savings for patients with CVDs, concluding that it is more cost-effective than standard of care (SoC) [21]. This mirrors the broad findings of 2 other systematic reviews covering a diverse range of DHIs [22,23]. However, 2 reviews were more focused on CVDs than on HF [21,22], with 1 review not including search terms related to HF [21]; 2 reviews focused on either economic models or randomized controlled trials (RCTs) but not both [22,23]; and 2 reviews only considered a limited range of DHIs [21,23]. To date, no comprehensive systematic review has been conducted to evaluate the economic evaluations of DHIs specifically in patients with HF, considering evidence from both models and RCTs.

### Objectives

The aim of this systematic review was to provide an overview and summarize published economic evaluations of DHIs in patients with HF that consider both models and analyses conducted alongside trial-based evaluations. Demonstrating the cost-effectiveness of DHIs will contribute to a better understanding of the potential economic implications of adopting these approaches.

## Methods

This systematic review was conducted in accordance with the PRISMA (Preferred Reporting Items for Systematic Reviews and Meta-Analysis) guidelines [24], and the review was registered in PROSPERO [25]. The PRISMA checklist is provided in Multimedia Appendix 1.

### Search Strategy

A systematic search was performed across 3 major electronic databases (PubMed, EBSCOhost, and Scopus) to identify economic evaluations of DHIs for patients with HF. Medical Subject Headings terms and text words related to “heart failure,” “digital health,” and “economic evaluation” were used to search from database inception to July 2023. Terms were combined using “OR” and “AND.” Full details are provided in Multimedia Appendix 2.

### Study Selection

The search results were exported to Mendeley Reference Manager (Elsevier Ltd) and checked for duplicates. Two reviewers (NZ and DM) independently performed a full-text review of the chosen articles after the preliminary title and abstract screening, using the inclusion and exclusion criteria detailed in Textbox 1.

Any disagreements were discussed, and a third reviewer was consulted for arbitration to arrive at a consensus if required. References were also searched for further relevant papers during the full-text reviews.

**Inclusion and exclusion criteria.**

**Inclusion criteria**

- Type of study: a full economic evaluation of digital health interventions (DHIs) for the management of patients with heart failure (HF), categorized as cost-benefit analysis, cost-utility analysis, cost-effectiveness analysis, and cost-minimization analysis
- Intervention: any DHI for patients with HF comprising a digital intervention for transmitting medical information to improve patients’ health status (DHIs are a broad concept encompassing eHealth, which refers to the application of information and communications technology in support of health and health-related fields; this includes the use of mobile devices such as smartphones and patient monitoring devices in medical and public health practices, commonly known as mobile health. DHIs also comprise emerging domains such as advanced computing sciences in big data, genomics, and artificial intelligence [13,26]. Standard of care was defined as the standard multidisciplinary management program [19,20], which includes regular planned follow-up for the purpose of safety and optimal drug dosing (standard of care with or without drug or exercise prescription), early detection of decompensation, and impact on disease progression that requires modification of the intervention or treatment regimen)
- Participants: adult patients with HF (aged ≥18 y)
- Time limits: searches were conducted for relevant articles published from the beginning of database entries to July 2023

**Exclusion criteria**

- Non-English studies, experimental and observational studies without economic evaluation, studies that did not report outcomes specific to HF, reviews, conference abstracts, and editorials

### Data Extraction

Two reviewers conducted data extraction from the full-text articles independently using a predetermined form covering general study characteristics (author, country, and year of publication), study design (type of economic evaluation, perspective, model type, time horizon, discount rate, intervention vs comparator, outcome measures, and sensitivity analysis), primary outcomes, and quality of reporting. Only results related to DHIs for patients with HF were extracted when many interventions were evaluated. The primary outcomes collected were the cost-benefit ratio, cost savings, and cost-effectiveness of DHIs. Cost-effectiveness is represented by the incremental cost-effectiveness ratio (ICER) per quality-adjusted life year (QALY) gained or per intermediate outcome measure such as mortality or hospitalization.

### Ethical Considerations

As we exclusively used published studies for this systematic review and did not involve patients or the public or conduct any patient interviews, a review by, or approval from, an institutional review board was not required.

### Quality of Reporting

The CHEERS (Consolidated Health Economic Evaluation Reporting Standards) checklist was used to assess the reporting quality of each study [27]. The CHEERS checklist includes 28 items, with 1 point assigned to each item when the quality criterion is fulfilled (and 0 points for not entirely conforming to the relevant criterion) to generate a total score, with 28 (representing 100%) being the maximum score. On the basis of the scores, studies are classified into 4 quality categories: excellent (score: 100%), good (score: 75%-99%), moderate (score: 50%-74%), and low (score: ≤49%) [28]. This reflects reporting quality rather than a view of overall importance or methodological quality.

### Analysis and Presentation of Results

The results are presented in a range of narrative tables by study. The included studies were categorized by the device or technology used for delivering DHIs in managing patients with HF (ie, noninvasive remote monitoring devices, telephone support, mobile apps and wearables, remote monitoring follow-up in patients with implantable medical devices, and videoconferencing systems) [13,17]. Secondary categorization separates results by model-based and RCT-based studies. Money values were converted to 2023 US dollars using the Campbell and Cochrane Economics Methods Group–Evidence for Policy & Practice Information Centre Cost Converter [29]. If the study did not specify the costing year, publication year was assumed to be the year of costing. A 3×3 permutation matrix shows how each intervention’s outcomes (improved, worsened, or unchanged) and costs (increased, decreased, or unchanged) compare with those of its comparator in the studies [30]. This permutation matrix also splits the findings by DHI type.

## Results

### Study Identification

The initial search retrieved 365 studies, of which 7 (1.9%) duplicates were excluded. Of the 358 studies left, the title and abstract screening process excluded 296 (82.7%). After a full-text screening of the remaining 62 studies, we excluded 37 (60%; 21/37, 57% were classified as partial economic evaluations, such as cost analysis, containing only descriptions of costs; 10/37, 27% did not report outcomes specifically for HF; and 6/37, 16% were conference abstracts), resulting in 25 (40%) out of 62 studies for inclusion in the analysis. Two extra studies were identified from reference reviews; thus, 27 studies [31-57] were included in this systematic review.

**Figure 1:**
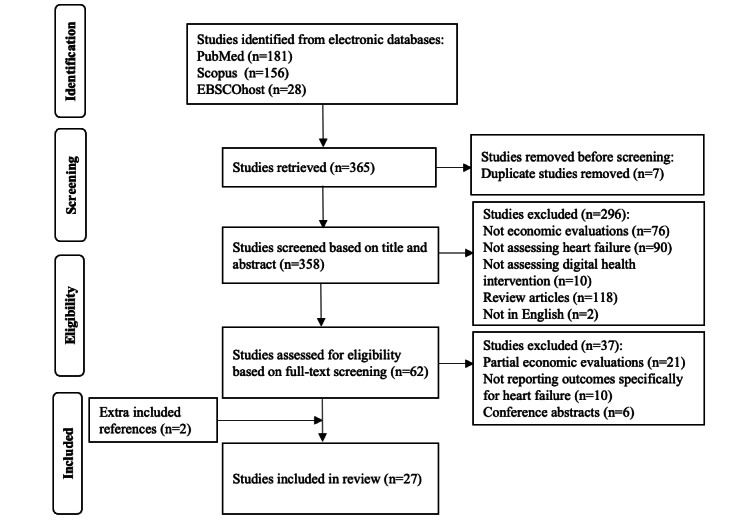
The PRISMA (Preferred Reporting Items for Systematic Reviews and Meta-Analyses) flowchart of the study selection process.

The selection process and flow diagram for the identification of studies are depicted in Figure 1.

### Study Characteristics and Design

Table 1 summarizes the general characteristics of the included studies. Of the 27 studies, 13 (48%) were conducted using a decision analytical model [31-35,40,47-51], 13 (48%) used trial-based data [36-39,41-46,54-57], and 1 (4%) used a combination of both [41]. The majority of the studies (25/27, 93%) were from HICs. Of the 27 studies, 6 (22%) were from the United States [34,43,44,51-53]; 3 (11%) each from the Netherlands [33,37,40] and the United Kingdom [32,35,50]; 2 (7%) each from Germany [36,38], Brazil [41,45], Canada [42,48], Italy [55,56], and Spain [46,49]; and 1 (4%) each from Australia [57], Poland [54], France [31], Hong Kong [47], and Denmark [39]. Of the 27 studies, 24 (89%) conducted a cost-utility analysis with cost and QALY as the outcome measures [31-35,37-40,42-51,54,56,57], 2 (7%) conducted a cost-effectiveness analysis (1/2, 50% with hospital readmission as the outcome measure [41] and 1/2, 50% with number of days alive and neither in hospital nor in inpatient care as the outcome measure [36]), and 1 (3%) conducted a cost-minimization analysis [55].

**Table 1:** General characteristics of included studies.

Study characteristics		Studies (n=27), n (%)
**Type of economic evaluation**		
	Cost-utility analysis	24 (89)
	Cost-effectiveness analysis	2 (7)
	Cost-minimization analysis	1 (4)
**Year of publication**		
	Before 2010	2 (7)
	2011-2015	6 (22)
	2016-2020	12 (45)
	2021-2023	7 (26)
**Region**		
	Europe	15 (56)
	North and South America	9 (33)
	Asia Pacific	3 (11)
**Perspective**		
	Health care system	16 (59)
	Health care provider	4 (15)
	Health care system and societal	4 (15)
	Societal	3 (11)
**Study type**		
	Model based	13 (48)
	Randomized controlled trial based	13 (48)
	Combination	1 (4)
**Time horizon**		
	Nonlifetime	15 (56)
	Not stated	6 (22)
	Lifetime	6 (22)
**Outcome measures**		
	Quality-adjusted life years	24 (89)
	Other effects	2 (7)
	Not stated	1 (4)
**Funding**		
	Nonprivate	15 (56)
	Not stated	7 (26)
	Private	3 (11)
	No funding	2 (7)

Of the 27 studies, 15 (56%) used a time horizon of >1 year (3/15, 20% were RCTs) [31-35,40,44,45,47,48,50-54]; the time horizon in 6 (22%) studies was ≤1 year (all were RCTs) [36,39,41-43,46]; 6 (22%) did not state the time horizon (5/6, 83% were RCTs and 1/6, 17% was model based) [37,38,49,55-57]. Of the 27 studies, 15 (56%) received grants from public organizations [31,32,35,36,38-41,43,44,46,49,54,56,57], 3 (11%) received funding from industry [37,50,51], 2 (7%) received no funding [42,55], and 7 (26%) did not declare their funding source [33,34,45,47,48,52,53].

A little more than half of the studies (14/27, 52%) measured effectiveness with HRQoL (or utilities) using a patient-based EQ-5D instrument [31,32,34,35,37-40,48,50-53,56,57]. Other studies used the generic Short Form Health Survey-36 with norm-based scoring [42,44,54], the Minnesota Living with Heart Failure Questionnaire [45], or a combination of both [36,43]. All studies included the direct costs of DHIs, such as the costs of the DHIs, inpatient and outpatient costs, monitoring and follow-up costs, and medication costs. Some of the studies (12/27, 44%) included nonmedical direct costs, such as travel and transportation costs [33,35,38,41,43,46,50,55]. Studies that used a societal perspective (6/27, 22%) also included indirect costs, such as productivity losses [33,38,43,46,55,56]. Details on the perspectives and included costs are provided in Multimedia Appendix 3 [19,20,31-57].

### Cost-Effectiveness of Devices or Technologies Used for Delivering DHIs in the Management of Patients With HF

#### Overview

This subsection describes the nature of the DHIs assessed for cost-effectiveness and presents cost-effectiveness findings by type of DHI in order of the number of studies identified. Details on summaries and outcomes from the studies are provided in Table 2; and relative costs, effects, and main outcomes are presented in Table 3. Overall, of the 27 studies, 24 (89%) found the DHIs to be cost-effective [31-34,37-43,45-57], whereas 3 (11%) were not cost-effective, particularly home telemonitoring (HTM) and telephone support [35,36,44].

**Table 2:** Summary and quality assessment of the included studies.

Study				Country		Time horizon		Discount rate (%)		ICER^a^ (in 2023 US $)		WTP^b^ threshold (in 2023 US $/QALY^c^)		Quality of reporting (CHEERS^d^ checklist)
**Noninvasive remote monitoring devices (n=9)**														
	**Model based**													
		Caillon et al [31], 2022	France		10 y		2.5		8456/QALY 5955/LY^e^		15,372		Excellent (score: 28/28, 100%)	
		Thokala et al [32], 2013	United Kingdom		30 y		3.5		20,715/QALY		34,895		Good (score: 25/28, 89%)	
		Albuquerque de Almeida et al [33], 2022	Netherlands		Lifetime		4		SoC^f^ vs HTM^g^: 45,277/QALY SoC vs HTM+DA^h^: 36,422/QALY		105,146		Good (score: 24/28, 86%)	
		Jiang et al [34], 2020	United States		35 y		3		40,691/QALY 37,641/QALY 106,837/QALY		53,183		Moderate (score: 21/28, 75%)	
		Thokala et al [35], 2020	United Kingdom		Lifetime		3.5		72,028/QALY		29,904		Good (score: 22/28, 79%)	
	**RCT^i,j^ based**													
		Völler et al [36], 2022	Germany		1 y		—^k^		−1474/d		—		Moderate (score: 20/28, 71%)	
		Boyne et al [37], 2013	Netherlands		—		N/A^l^		59,822/QALY		74,182		Moderate (score: 19/28, 68%)	
		Sydow et al [38], 2021	Germany		—		—		Dominant (cost savings: 2358 per patient year)		—		Good (score: 21/28, 75%)	
		Vestergaard et al [39], 2020	Denmark		1 y		4		8020/QALY		31,064		Good (score: 25/28, 89%)	
**Telephone support (n=7)**														
	**Model based**													
		Grustam et al [40], 2018	Netherlands		20 y		4		UC^m^ vs HTM: 17,597/QALY UC vs NTS^n^: 11,661/QALY		112,811		Good (score: 25/28, 89%)	
	**RCT based**													
		Ruschel et al [41], 2018	Brazil		6 mo		N/A		PHS^o^ framework: 332 per hospital readmission prevented; the private health care system, using a perspective of private health care system, the intervention was dominant (cost saving)		—		Good (score: 25/28, 89%)	
		Cui et al [42], 2013	Canada		1 y		N/A		3331/QALY		55,985		Good (score: 24/28, 86%)	
		Hebert et al [43], 2008	United States		1 y		N/A		Societal: 26,273/QALY Payer: 5500/QALY		37,441		Good (score: 23/28, 82%)	
		Smith et al [44], 2008	United States		18 mo		—		212,586/QALY		144,744		Good (score: 21/28, 75%)	
		Bocchi et al [45], 2018	Brazil		Mean 2.47 (SD 1.75) y		—		4114/QALY		10,825		Moderate (score: 20/28, 71%)	
		Gonzalez-Guerrero et al [46], 2018	Spain		1 y		5		Health care: 6611/QALY Societal: 43,856/QALY		76,002		Good (score: 24/28, 86%)	
**Remote monitoring follow-up in patients with implantable medical devices (n=7)**														
	**Model based**													
		Cowie et al [50], 2017	United Kingdom		10 y		3.5		31,177/QALY		32,351		Good (score: 25/28, 89%)	
		Schmier et al [51], 2016	United States		5 y		3		50,571/QALY		112,993		Good (score: 21/28, 75%)	
		Sandhu et al [52], 2016	United States		Lifetime		3		82,282/QALY		172,712		Good (score: 21/28, 75%)	
		Martinson et al [53], 2017	United States		5 y		3		13,855/QALY		56,496		Good (score: 23/28, 82%)	
	**RCT based**													
		Niewada et al [54], 2021	Poland		Lifetime		3.5		56,333/QALY		91,300		Good (score: 21/28, 75%)	
		Calò et al [55], 2013	Italy		—		—		—		—		Moderate (score: 16/28, 57%)	
		Zanaboni et al [56], 2013	Italy		—		—		Intervention dominant		62,166		Moderate (score: 20/28, 71%)	
**Mobile apps and wearables (n=3)**														
	**Model based**													
		Jiang et al [47], 2021	Hong Kong, Special administrative region, China		10 y or until death, whichever occurred first		3		4380/QALY		49,949		Good (score: 21/28, 75%)	
		Boodoo et al [48], 2020	Canada		25 y		1.5		7127/QALY		40,119		Good (score: 25/28, 89%)	
		Cano Martin et al [49], 2014	Spain		—		3		16,064/QALY		—		Good (score: 21/28, 75%)	
**Videoconferencing system (n=1)**														
	**RCT based**													
		Hwang et al [57], 2018	Australia		—		—		−3325/QALY (savings)		40,000		Good (score: 24/28, 86%)	

^a^ICER: incremental cost-effectiveness ratio.

^b^WTP: willingness-to-pay.

^c^QALY: quality-adjusted life year.

^d^CHEERS: Consolidated Health Economic Evaluation Reporting Standards.

^e^LY: life-year.

^f^SoC: standard of care.

^g^HTM: home telemonitoring.

^h^DA: diagnostic algorithm.

^i^RCT: randomized controlled trial.

^j^RCT-based evaluation extended with a decision tree model (combination).

^k^Not stated.

^l^N/A: not applicable.

^m^UC: usual care.

^n^NTS: nurse telephone support.

^o^PHS: public health care system.

**Table 3:** Relative costs, effects, and main outcomes.

Relative cost	Relative effect		
	−^a^	0^b^	1^c^
−	No study	DHI^d^ is not cost-effective (1/27, 4%) Noninvasive remote monitoring devices SoC^e^+interactive bidirectional HTM^f^ system (Motiva) vs SoC and patient diary to document health issues once a week [36]^g^	DHI is cost-effective (15/27, 56%) Noninvasive remote monitoring devices SCAD^h^, home-based interactive telemonitoring service vs standard hospital-based care [31]^i^ STSHM^j^ interface+STSHH^k^ contact+HTM vs SoC [32]^i^ HTM+DA^l^ vs SoC or HTM+DA vs HTM only [33]^i^ Universal SoC+HTM for NYHA^m^ class II to IV and class III to IV vs SoC [34]^i^ Telephone support HTM or nurse telephone support vs SoC+patient evaluation at the clinic every 4 months [40]^i^ Nurse-led home visit vs regular visit to outpatient clinic [41]^g,n^ HL^o^ (nurses and health care providers providing telephone support)+SoC or HL+in-house monitoring+SoC vs SoC [42]^g^ Nurse-led program vs SoC [43]^g^ Mobile apps and wearables Add-on HTM via app vs SoC [47]^i^ HTM system (Medly) via app vs SoC, including specialized multidisciplinary HF^p^ clinics [48]i Remote monitoring follow-up in patients with implantable medical devices Implantable hemodynamic sensor (CardioMEMS HF system) vs implantable usual care [50]^i,q^ Implantable hemodynamic sensor (CardioMEMS HF system) vs implantable usual care [51]^i,q^ Implantable hemodynamic sensor (CardioMEMS HF system) vs implantable usual care [52]^i,q^ Implantable hemodynamic sensor (CardioMEMS HF system) vs implantable usual care [53]^i,q^ HCTR^r^, including telecare, telerehabilitation, and implantable+SoC vs SoC only [54]^g^ DHI is not cost-effective (2/27, 7%) Noninvasive remote monitoring devices HTM vs SoC [35]^i^ Telephone support DM^s^ (telephone support+augmented HTM) vs SoC [44]^g^
0	No study	DHI is cost-effective (1/27, 4%) Noninvasive remote monitoring devices HTM vs SoC [37]^g^	N/A^t^
1	No study	DHI is cost saving (2/27, 7%) Remote monitoring follow-up in patients with implantable medical devices ICD^u^ follow-up vs quarterly in-hospital follow-ups [55]^^g^^ Videoconferencing system Web-based telerehabilitation vs in-person center-based program [57]^g^	DHI is dominant (6/27, 22%) Noninvasive remote monitoring devices Additional noninvasive structured RPM^v^ vs SoC [38]^g^ HTM with a telekit (consisting of a tablet, a digital blood pressure monitor, and a scale) vs SoC [39]^g^ Telephone support HTM via telephone follow-up vs SoC [45]^g^ DMP^w^ vs postdischarge SoC [46]^g^ Mobile apps and wearables CardioManager app vs SoC [49]^i^ Remote monitoring follow-up in patients with implantable medical devices Wireless transmission–enabled ICD vs scheduled in-person evaluations [56]^g^

^a^Digital health intervention has lower cost or lower effectiveness than the comparator.

^b^0: digital health intervention has the same cost and same effectiveness as the comparator.

^c^1: digital health intervention has a higher cost or higher effectiveness than the comparator.

^d^DHI: digital health intervention.

^e^SoC: standard of care (as defined by the European Society of Cardiology and the American Heart Association, American College of Cardiology, and Heart Failure Society of America, it is the standard multidisciplinary management program, which includes regular planned follow-up for the purpose of safety and optimal drug dosing [standard of care with or without drug or exercise prescription], early detection of decompensation, and impact on disease progression that requires modification of the intervention or treatment regimen).

^f^HTM: home telemonitoring.

^g^Randomized controlled trial based.

^h^SCAD: Suivi Clinique A Domicile (Clinical Follow-Up At Home).

^i^Model based.

^j^STSHM: structured telephone support via human-to-machine.

^k^STSHH: structured telephone support via human-to-human.

^l^DA: diagnostic algorithm.

^m^NYHA: New York Heart Association.

^n^Randomized controlled trial–based evaluation extended with a decision tree model (combination).

^o^HL: Health Lines.

^p^HF: heart failure.

^q^Implantable usual care described as patients with the device implanted but where the data were not used to guide management for remote monitoring.

^r^HCTR: hybrid comprehensive telerehabilitation.

^s^DM: disease management.

^t^N/A: not applicable.

^u^ICD: implantable cardioverter defibrillator.

^v^RPM: remote patient management.

^w^DMP: Disease management program.

#### Noninvasive Remote Monitoring Devices

Noninvasive remote monitoring devices (n=9) assessed for cost-effectiveness included HTM using medical devices and digital tablets. These devices enable the monitoring of a patient’s vital parameters at home, including weight, blood pressure, heart rate, and heart rhythm. These devices enable the transmission of physiological data to the health care team, allowing for early detection of deterioration in patients with HF [31-39]. The prompt sending of these data to health care professionals for assessment facilitates the timely identification of significant changes and enables early interventions [37,38]. Early interventions help prevent complications and enable patients to avoid emergency admissions, improving patient outcomes [31,32,39].

Most of the economic evaluations (7/9, 78%) of noninvasive remote monitoring devices were compared to SoC [19,20] as defined in the international guidelines [31,33-35,38,39]. Although the definitions of SoC are similar, some of the studies (2/9, 22%) provided additional details regarding the follow-up procedures, such as SoC with follow-up once a week [36] or 4 preplanned outpatient clinic visits [37]. Among the 9 studies, 4 (44%) used Markov models [31,32,34,35], 1 (11%) used a patient-level discrete-event simulation model [33], and 4 (44%) were trial based [36-39]. Some of the economic evaluations (4/9, 44%) showed that the implementation of noninvasive remote monitoring devices requires extra costs, mainly regarding the cost of HTM for HF management [31-34]. Nonetheless, the use of this technology was also accompanied by improved outcomes, such as improved HRQoL [31,32,34,35,37-39]. Although the majority of the results suggested that DHIs were cost-effective, the findings were conflicting. Although most of the studies (7/9, 78%) indicated that noninvasive remote monitoring devices for managing patients with HF were generally cost-effective [31-34,37-39], 22% (2/9) found dissimilar results: of these 2 studies, 1 (50%) conducted from the UK health care perspective reported that the incremental cost per QALY gained for HTM using noninvasive remote monitoring devices exceeded the acceptable willingness-to-pay (WTP) thresholds [35], while 1 (50%) conducted in Germany concluded that remote monitoring had higher costs and worse outcomes than SoC and was therefore not an efficient option [36].

#### Telephone Support

Structured telephone support (n=7), defined in the included studies, refers to the provision of HTM through self-care support or management by health care professionals, such as nurses, through regular telephone calls, typically on a monthly basis [42,43,45,46]. Of the 7 economic evaluations that used telephone support, 6 (86%) were based on RCTs [41-46], and 1 (14%) was model based [40]. The primary objective of telephone support includes assessing symptoms, reviewing current medications, and providing timely feedback to both physicians and patients [42]. The length of the intervention ranged from 4 to 30 months. The extra costs associated with the telephone support intervention compared to SoC included the costs of telephone calls and specialist follow-up visits. The outcomes measured included hospital readmission prevented over 24 weeks [41] and HRQoL [40,42,44-46]. The comparator SoC adhered to the definition provided in the guidelines [19,20], or it involved routine ambulatory evaluations in 3 to 4 months [45]. Overall, the results showed that telephone support was cost-effective compared to SoC (6/7, 86%) [40-43,45,46]. However, 1 (14%) of the 7 studies concluded that telephone support was not cost-effective in the United States because it surpassed the acceptable cost-effectiveness threshold as higher total costs in the intervention group were combined with a relatively small difference in health outcomes compared to the SoC group (ie, usual management by physicians) [44].

#### Remote Monitoring Follow-Up in Patients With Implantable Medical Devices

Of the 27 studies, 7 (26%) assessed the cost-effectiveness of remote monitoring follow-up in patients with implantable medical devices. Of these 7 studies, 3 (43%) were trial based [54-56], and 4 (57%) were model based [50-53]. The interventions included remote monitoring follow-up for patients using cardiac implantable electronic devices, which are used to manage conditions such as bradycardia and HF to prevent sudden cardiac death [58]. The 4 model-based studies [50-53] assessed the same device, that is, the CardioMEMS implantable hemodynamic sensor, which provides remote real-time pressure measurements from the pulmonary artery [59]. This wireless sensor transmits hemodynamic information to the patient database website, enabling health care professionals to promptly make decisions regarding treatment initiation and adjustments when changes in pulmonary artery pressure and signs of HF are detected. The comparator comprised usual care described as patients with the device implanted but where the data were not used to guide management for remote monitoring (implantable usual care) [50-53].

The 3 trial-based studies focused on remote monitoring follow-up with patients having cardiac implantable electronic devices compared to conventional follow-up [54-56]. The comparator included patients who typically attended regular follow-up visits at the clinic based on a predetermined calendar schedule [54]. Furthermore, 2 (67%) of these 3 studies provided specific details of their study settings: of these 2 studies, 1 (50%) described outpatient clinic visits every 3 to 6 months according to the standard schedule at the participating center [55], while 1 (50%) had scheduled in-office visits at 4, 8, 12, and 16 months [56]. Generally, costs associated with implantable devices followed by remote monitoring consist of the cost of visits to physicians and nurses and fees for the remote monitoring service, as well as the costs of the transmitter device, battery replacement, and cardiovascular treatment.

All studies indicated that implantable medical devices, especially for patients with severe HF (eg, New York Heart Association class III and class IV), were considered cost-effective [50-56]. In Italy, they were even deemed a dominant strategy, leading to improved health outcomes while incurring lower total costs [56].

#### Mobile Apps and Wearables

Of the 27 studies, 3 (11%) assessed the cost-effectiveness of providing HTM through expert counseling services via mobile apps [47-49]. The mobile apps provide a platform for patients to self-manage their heart condition [48]. The information section in the app contains a patient manual and medical information [49], while a separate section enables users to track their activity (physical activity and food consumption) and record health measurements such as vital signs [47-49]. In addition, the app includes a medication registry feature that allows patients to set reminders for medication administration times [49]. The features of mobile apps in the included studies were similar, that is, they included a feature that allowed the patient to transmit vital measurements (heart rate, blood pressure, and weight) daily to the HF management team, followed by interpretation by experts and categorization of the patient’s condition as well as feedback regarding the patient’s condition such as medication dosage adjustments or recommendations for the patients to visit the emergency department. Reminders for patients to enter data were in the form of alarms [48,49]. All studies in this group conducted a model-based economic evaluation that compared add-on mobile apps to SoC [19,20]. The additional costs of mobile app technology rely mainly on monitoring and treatment, with outcomes captured as HRQoL. All included studies indicated that the mobile apps were cost-effective (ie, below the WTP thresholds in each setting) [47-49].

#### Videoconferencing System

Only 1 (4%) of the 27 studies assessed the cost-effectiveness of providing specialist consultation services to remote patients with HF via a videoconferencing system (known as telerehabilitation) [57]. A web-based commercial videoconferencing platform was used for synchronized audiovisual communication with groups of up to 4 participants [57]. The videoconferencing system equipment included a laptop computer and mobile broadband devices connected to 3G wireless broadband internet; in addition, the participants were provided a finger pulse oximeter, an automatic sphygmomanometer, free weights, and resistance bands [57]. In telerehabilitation, a physiotherapist supervised each training session, and a physiotherapist and a nurse led the information session [57]. The results suggested no significant differences in QALYs, but the health care costs per participant were significantly lower in the telerehabilitation group, with a savings of US $3325 per QALY [57].

### Quality of Reporting

Of the 27 studies, 20 (74%) were rated *good* [32,33,35,38-44,46-54,57], 6 (22%) were rated *moderate* [34,36,37,45,55,56], and 1 (4%) was rated *excellent* [31]. Table 2 shows the percentage of items fulfilled by each study according to the CHEERS checklist.

The degree of adherence to the reporting criteria in the CHEERS checklist varied across the sections. Some items, such as background, intervention and comparator, study findings, generalizability, and funding, were adequately reported by all studies. The CHEERS checklist emphasizes the inclusion of essential and specific elements in the methods section, and nearly all studies included in this analysis comply with the checklist requirements, for example, the measurement and valuation of resources and costs (26/27, 96%) [31-33,35-57], perspective (26/27, 96%) [31-49,51-57], setting and location (25/27, 93%) [31-43,45-50,52-57], the measurement of outcomes (25/27, 93%) [31-36,38-48,50-57], and the selection of outcomes (24/27, 89%) [31-43,45-48,50-54,56,57]. However, there were certain items in the methods section that were reported less often; for instance, the approach to engagement with patients and other individuals affected by the study was only addressed in 2 (7%) of the 27 studies [31,39], and the impact of such engagement was discussed in only 1 (4%) of the 27 studies [31]. This limited reporting may be due to the fact that these items apply specifically to evaluation from trial-based data.

## Discussion

### Principal Findings

This systematic review comprehensively searched for, and summarized, the economic evaluations of various DHI devices used for managing patients with HF. In this review, we identified 27 studies, including both RCT- and model-based economic evaluations. The findings indicated that the types of DHI devices that were most frequently subjected to an economic evaluation were noninvasive remote monitoring devices (eg, HTM using digital tablets) and medical devices that enabled the transmission of physiological data, followed by telephone support, mobile apps and wearables, remote monitoring follow-up in patients with implantable medical devices (eg, implantable cardioverter defibrillators), and a videoconferencing system. The 27 studies, except for 2 (7%) from Brazil, were conducted in HICs, highlighting the lack of such assessment in LMICs. Despite the diverse range of devices and technologies used for delivering the interventions, the overall results demonstrated that DHIs are potentially more cost-effective than non-DHI alternatives or SoC.

Our findings suggest that HTM via mobile apps and wearables [47-49], home-based telerehabilitation using a videoconferencing system [57], and remote monitoring follow-up in patients with implantable medical devices [50,51,54-56] may be potentially dominant options in managing HF, with less total cost and higher effectiveness. The included studies demonstrate that remote monitoring follow-up in patients with implantable devices resulted in increased coverage of patient services, improved HRQoL [50-54,56], reduced years of life lost, and potentially reduced cost [55,56]. Of the 7 studies in this category, 4 (57%) focused on the economic evaluation of remote monitoring of intracardiac and pulmonary artery pressures in patients with HF via implantable hemodynamic monitoring devices in the United States and the United Kingdom [50-53]. The ICERs ranged from US $13,855 to US $82,782, and all were estimated to be below the respective WTP thresholds in each setting and deemed cost-effective. The subgroup analysis estimated that such a device might be more beneficial in terms of cost-effectiveness in patients with both types of HF: those with reduced ejection fraction and those with preserved ejection fraction [52]. This finding aligns with the updated guidelines recommending the consideration of monitoring pulmonary artery pressures using a wireless hemodynamic monitoring system, particularly for patients with symptoms of HF, to enhance clinical outcomes [20].

Nevertheless, the evidence is limited, especially in mobile apps and wearables as well as home-based telerehabilitation. While mobile apps and wearables are occasionally marketed directly to consumers for health and lifestyle maintenance, of the 27 studies, 3 (11%) focused on assessing HTM through HF-specific apps. The limited evidence and lack of clear app standards pose challenges for decision-makers to make recommendations, although the understanding of the importance of assessment and regulation regarding these DHIs is currently growing. All comparators (3/3, 100%) in these interventions consisted of SoC, as defined in the guidelines [19,20], which improves the generalizability of the findings.

Furthermore, most base-case findings concerning HTM via noninvasive remote monitoring devices and telephone support indicate that HTM is more costly but more effective than conventional SoC comparators. The observed ICERs ranged from US $8020 [39] to US $106,837 [34] for noninvasive remote monitoring devices and from US $3331 [42] to US $212,586 [44] for telephone support, both per QALYs gained. While most of the studies (9/16, 56%) concluded that the ICERs remained below the WTP thresholds in their respective settings, thus making them cost-effective, certain countries with lower WTP thresholds yielded different findings (3/16, 19%), resulting in conflicting conclusions regarding the cost-effectiveness of these interventions. The WTP thresholds identified in the studies involving noninvasive remote monitoring devices ranged from US $15,372 [31] to US $105,146 [33], and all were derived from studies conducted in HICs. Hence, it is crucial to be cautious when applying these results in wider settings, especially in LMICs with relatively lower WTP-per-QALY threshold levels, because what may be considered a cost-effective intervention in settings with higher WTP thresholds could yield different outcomes in countries with lower WTP thresholds. Other included studies (4/16, 25%) indicated that HTM via noninvasive remote monitoring devices or telephone support can be a dominant strategy [38,39,45,46], with a lower total cost and higher effectiveness than SoC. The cost savings observed in these studies were primarily attributed to reduced hospitalization expenses, especially with regard to the noninvasive devices. This aligns with the primary objective of intervention in HF management because lowering cardiovascular-related hospitalizations and all-cause mortality represents an important clinical end point in most trials assessing HF treatments [60]. The effectiveness of HTM [32] and the cost of HF management [31] are identified as some of the most sensitive parameters that could influence the outcomes of the base-case analysis in model-based studies. Moreover, input parameters associated with hospitalization [33,34,41] and the cost of the intervention [41,44,45] are among the most sensitive variables in RCT-based studies.

In the analysis using noninvasive remote monitoring devices, the distribution of model-based and RCT-based studies is comparable and primarily reflects current updates because the majority (7/9, 78%) were published recently. In the case of telephone support, the economic evaluations are predominantly based on RCTs, with the most recent study dating back to 2018 [41,45,46]. The international guidelines from both the European Society of Cardiology and the American College of Cardiology written in the era before the COVID-19 pandemic do not recommend routine use of remote monitoring or HTM [61,62]. However, during and after the pandemic, the updated version of guidelines for the treatment and management of HF highlights the potential benefits of continuous monitoring of clinical parameters and optimizing care. Despite inconsistencies in its comparative effectiveness and cost-effectiveness, HTM is mentioned as a possible means of monitoring patients [19,20]. Previous evidence indicated that systems that focus on a health maintenance approach through continuous optimization by using DHIs such as noninvasive HTM and telephone support seem to reduce the risk for hospitalizations and all-cause mortality and subsequently improve HRQoL [63,64].

Our findings indicate inconsistencies in the cost-effectiveness of DHIs, which might be attributed to intervention variation. This variation makes it challenging to compare the different DHIs in terms of design, effectiveness, and cost-effectiveness. We stratified the findings by device, which allows a comparison of each technology and provides a better understanding of the cost and effectiveness of adopting DHIs. Economic evaluations of DHIs pose unique challenges compared to those of drugs and medical devices, primarily due to their interacting and evolving features. As observed in this review, most published economic evaluations of DHIs adhere to standard methodological recommendations for evaluating health care technologies, such as pharmaceutical drugs and medical devices. However, there is an argument that these methodological assumptions may not fully reflect the distinct nature of DHIs, which are typically complex interventions composed of multiple interacting components. Consequently, assessing their cost-effectiveness requires a broader evaluation of costs and effects. This evaluation should extend beyond using just 1 outcome measure, such as HRQoL, to include nonhealth benefits and costs beyond health care [65].

In this review, we observed that the incorporation of DHIs is generally associated with improved effectiveness, despite incurring higher total costs. Both short- and long-term time horizons were used in the included studies. The studies demonstrated improved cost-effectiveness of DHIs with a long-term time horizon (≥5 y), indicating the importance of considering a sufficient time horizon to assess the impact of the technology on outcomes. Economic evaluations conducted alongside RCTs tend to use a short time horizon, in line with the timeline of the trials. Determining the time horizon in economic evaluations is crucial because it determines the timing of costs and benefits and how long they should be spread out. When evaluating technology for patients with chronic conditions with long-term potential effects on both cost and health outcomes, assessments with a time horizon of ≤1 year may not consider benefits spread out over extended periods, potentially resulting in an underestimation of its cost-effectiveness. Combining data from RCTs with modeling that allows the projection of costs and effectiveness in the coming years could offer a viable solution to estimate the economic evaluations of DHIs more accurately.

A previous systematic review on HTM or structured telephone support programs for patients with HF suggested that these interventions were considered cost-effective compared to SoC [66]. Similar to these results, according to the included studies in this review, DHIs are generally more cost-effective than standard postdischarge care for managing HF. DHI systems, using infrastructure such as the telephone and the internet, allow patients to access cardiac rehabilitation programs from home and report signs of worsening conditions, regardless of location or the time of day. Such systems also enable remote patient monitoring, reducing the burden on hospitals and health care resources and potentially leading to overall cost savings [67]. The widespread availability of internet and telephone access in patients’ homes, combined with the ease and affordability of implementing remote monitoring systems in clinical practice, make DHIs a potentially cost-effective option [15].

Given the significant clinical and economic burden of HF in LMICs [8], the potential implementation of DHIs in these settings is promising. However, evidence on the cost-effectiveness of DHIs in LMICs is very limited, as observed in this review. To ensure successful implementation, there is a need to test the validity and reliability of DHIs, tailoring their function and design to the specific needs of programs in LMICs, thereby minimizing potential implementation challenges [68]. The transformative potential of digital health in improving health outcomes depends on substantial investment in governance, institutional capacity, and workforce training to navigate the evolving digital landscape of health systems [69]. Comprehensive evidence on the acceptability and cost-effectiveness of DHIs within specific settings in LMICs, including financial considerations, must be integrated into routine health budgets and budgeting processes to assess full-scale sustainability. Consequently, securing sufficient and sustainable financial resources, especially given the financial constraints in LMICs, is crucial. Mobilizing additional resources from development partners is essential in this regard. With strategic investments aligned with national digital health strategies, digital health has the potential to enhance care efficiency and cost-effectiveness, ultimately leading to improved health care service delivery [70].

### Strengths and Limitations

The strength of our systematic review is that we assessed various DHIs—both decision-analytic model-based and trial-based economic evaluations of DHIs in managing HF globally—encompassing HTM, rehabilitation, and remote monitoring follow-up after cardiac device implantation. The results of this study may facilitate comparisons and assist policy makers in making informed decisions on how to improve the health outcomes of patients with HF.

Inevitably, our study has some limitations. Due to the variability of the methods, devices, and DHI technologies in the included studies, the comparability of studies is limited. We try to overcome this limitation by using a narrative approach; thus, the variations in methodology and study design can be observed thoroughly. It is important to note that nearly all included studies (25/27, 93%) are from HICs, and caution is warranted when generalizing their results, particularly to LMICs, due to differences in health care systems and resource availability. In addition, although we used a broad definition of DHIs that includes genomics for personalized medicine and artificial intelligence, we did not find any studies related to these concepts. This may be attributed to the existing gaps in clinical and cost-effectiveness evidence [71] when integrating these approaches in the context of HF. Nonetheless, the use of precision medicine, which holds the potential to improve clinical outcomes, represents a promising avenue for the future of precision medicine [72]. In addition, the search strategy used for this systematic review had some constraints. The search terms were constructed using the population, intervention, comparator, and outcomes method, emphasizing a predefined set of terms related to economic evaluations, HF, and DHIs. It is possible that this approach may have overlooked relevant studies that use different keywords. To mitigate this potential gap, we cross-checked the references of the included economic evaluations. Thus, even if we did overlook any, we anticipate that the number will be minimal. Furthermore, considering the variability in the DHIs, modeling approaches, ICER values, and WTP thresholds, it is crucial to perform economic evaluations customized to the specific setting and country. This is especially relevant for LMICs, where the choice of technology, analytical methods, and models should align with the local context.

### Conclusions

This review includes 27 studies—model based, RCT based, and combination of both—that focus on economic evaluations of DHIs for patients with HF. The results indicated that noninvasive remote monitoring devices, followed by telephone support, mobile apps and wearables, remote monitoring follow-up in patients with implantable medical devices, and videoconferencing systems are the DHI devices most frequently subjected to economic evaluations in managing HF. Our main findings suggested that adopting DHIs as part of HF treatment and management, in general, requires extra costs but is accompanied by improved health outcomes as measured by HRQoL, compared to SoC, thus seeming to be cost-effective. However, this depends on each country’s WTP thresholds for considering cost-effectiveness. The majority of the studies (25/27, 93%) are from HICs, and the findings may not be generalizable to LMICs. Improvement in the quality of reporting, especially in the methodology of further economic evaluations, would better inform the cost- and health-related outcomes of incorporating DHIs for patients with HF.

## Appendix

Multimedia Appendix 1 PRISMA (Preferred Reporting Items for Systematic Reviews and Meta-Analyses) 2020 checklist.

Multimedia Appendix 2 Search strategy.

Multimedia Appendix 3 Perspective, costs, and sensitivity analysis.

## Abbreviations

CHEERS: Consolidated Health Economic Evaluation Reporting Standards
CVD: cardiovascular disease
DHI: digital health intervention
HF: heart failure
HIC: high-income country
HRQoL: health-related quality of life
HTM: home telemonitoring
ICER: incremental cost-effectiveness ratio
LMIC: low- and middle-income country
PRISMA: Preferred Reporting Items for Systematic Reviews and Meta-Analyses
QALY: quality-adjusted life year
RCT: randomized controlled trial
SoC: standard of care
WTP: willingness-to-pay
