# Supplementary material for: Economic Evaluations of Digital Health Interventions for Patients With Heart Failure: Systematic Review
Source: J Med Internet Res. 2024 Apr 30;26:e53500. doi: 10.2196/53500 (PMC11094606; doi:10.2196/53500)
Supplement: Multimedia Appendix 2 [file jmir_v26i1e53500_app2.docx]

**Multimedia Appendix 2**

**Search Strategy**

Heart failure term:

"Heart Failure"[Mesh] OR “heart failure” OR “cardiac heart failure” OR “chronic heart failure” OR “congestive heart failure” OR “CHF”

Digital health term:

"Telemedicine"[Mesh] OR “telemedicine” OR “telemonitoring” OR “telehealth” OR “telerehabilitation” OR “telepharmacy” OR “mobile health” OR “tele*” OR “digital health”

Economic evaluation term:

"Cost-Benefit Analysis"[Mesh] OR “cost-minimization” OR “cost-effectiveness” OR “cost-utility” OR “cost-benefit”

PUBMED

(("Heart Failure"[Mesh] OR “heart fail*”[tw] OR “cardiac heart fail*”[tw] OR “chronic heart fail*”[tw] OR “congestive heart fail*”[tw] OR “CHF*”[tw]) AND ("Telemedicine"[Mesh] OR “telemedicine*”[tw] OR “telemonitoring*”[tw] OR “telehealth*”[tw] OR “telerehabilitation*”[tw] OR “telepharma*”[tw] OR “mobile health*”[tw] OR “tele*”[tw]) AND ("Cost-Benefit Analysis"[Mesh] OR “cost-minimization*”[tw] OR “cost-effectiveness*”[tw] OR “cost-utility*”[tw] OR “cost-benefit*”[tw]))

EBSCOhost and SCOPUS

((heart failure OR cardiac heart failure OR chronic heart failure OR congestive heart failure OR CHF) AND (telemedicine OR telemonitoring OR telehealth OR telerehabilitation OR telepharmacy OR mobile health) AND (cost minimization analysis OR cost utility analysis OR cost effectiveness analysis OR cost benefit analysis))
