# Supplementary material for: Economic Evaluations of Digital Health Interventions for Patients With Heart Failure: Systematic Review
Source: J Med Internet Res. 2024 Apr 30;26:e53500. doi: 10.2196/53500 (PMC11094606; doi:10.2196/53500)
Supplement: Multimedia Appendix 3 [file jmir_v26i1e53500_app3.docx]

**Multimedia Appendix 3**

Perspectives and Costs

| **Author, Year** | **Perspective** | **Costs data** | | |
| --- | --- | --- | --- | --- |
|  |  | **Direct costs** | | **Indirect costs** |
|  |  | **Medical** | **Non-medical** |  |
| Caillon et al, 2022 [31] | Collective perspective by the national health insurance (NHI), complementary private health insurance, and the patient | Cost of SCAD programme, Management cost for non-hospitalised patients, HF hospitalization cost, palliative care cost | N/A | N/A |
| Thokala et al, 2013 [32] | NHS in England and Wales | The costs of the device, monitoring costs and medical care costs, the hourly NHS staff rates | N/A | N/A |
| Albuquerque de Almeida et al, 2022 [33] | Societal | Hospitalization Costs, Drug Costs, Outpatient Visit Costs; cost of emergency room visits, office visits, home visits, and telephone calls; equipment and service fees and the instalment fee | Traveling Expenses | Informal Care Costs, Costs Related to Productivity Losses |
| Jiang et al, 2020 [34] | Healthcare provider | Cost of HF-related hospitalization (cost of inpatient care and LOS of patients); cost of HTM (smartphone, blood pressure monitor, weigh scale, licensing fee) | N/A | N/A |
| Thokala et al, 2020 [35] | Healthcare | Costs for general practitioner attendances, nurse visits, cost related to accident and emergency and hospital admissions (reported separately for HF and other causes), | N/A | N/A |
| Voller et al, 2022 [36] | Healthcare | Cost of inpatient and outpatient care, rehabilitation, nursing, medication, and life-saving appliances | N/A. | N/A |
| Boyne et al, 2013 [37] | Healthcare | The costs of hospitalisations and emergency room visits, GP, HFN, specialists, physiotherapist, psychological support and home care | N/A | N/A |
| Sydow et al, 2021 [38] | German statutory health insurance | the costs for hospital treatment [total hospitalizations, unplanned cardiovascular (CV) hospitalizations, and unplanned HF hospitalizations], outpatient treatment, therapeutic appliances, health care products, rehabilitation treatment, medications, home nursing care, | Transportation costs | sickness leave payments |
| Vestergaard et al, 2020 [39] | Danish Public healthcare | Cost of prescription medicine, cost of inpatient, outpatient and emergency hospitalisations, cost of personal care, practical help, home nursing, rehabilitation and telehealthcare activities | N/A | N/A |
| Grustam et al, 2018 [40] | Third-party payer | Telemonitoring cost (equipment and service fee; instalment fee); health care cost (nurse, GP, specialist, hospitalist, telephone call, telehealth nurse); hospital-related costs (day in a hospital, ER visit) | N/A | N/A |
| Ruschel et al, 2018 [41] | Brazilian Public Health care System (PHS) and private healthcare system | HF admission, HF-related ER visit, cost of nursing care | Transportation, telephone calls | N/A |
| Cui et al, 2013 [42] | Healthcare system | Family physician visits, physician specialist visits, cardiac physician visits, internist specialist visits and hospital in-patient days | N/A | N/A |
| Hebert et al, 2008 [43] | Societal and payer | Intervention cost (intervention materials, telephone service, nurse salaries, physician time over-seeing the nurse activity); medical costs (inpatient, outpatient, and ER) | Transportation cost | Cost of informal care (by friends, family members, or paid housekeepers) |
| Smith et al, 2008 [44] | Healthcare system | Cost of ER visits, inpatient admissions, inpatient and outpatient procedures, outpatient drugs, outpatient physician visits, laboratory work, and intervention costs | N/A | N/A |
| Bocchi et al, 2018 [45] | Payer | The cost of human resources, educational meetings, and monitoring call costs; cardiovascular and noncardiovascular hospital and emergency admission; emergency visits that did not require an overnight stay in the hospital; HF-related diagnostic and nonpharmacological therapeutic procedures; heart transplantation | N/A | N/A |
| Gonzalez-Guerrero et al, 2018 [46] | Societal and healthcare | Hospitalization (days), Specialist office visits, Follow-up visits at the GDH, Telephone contacts, PCP visits, Emergency visits, Rehabilitation (sessions), Medications | Transportation, Residential care (days) | Caregiver time |
| Cowie et al, 2017 [50] | Healthcare payer | Cost of an implant complication, total cost of implant procedure including cost of equipment and device, cost of a HF hospitalization, monthly cost to deliver medical care | N/A | N/A |
| Schmier et al, 2016 [51] | Payer | HF and non-HF hospitalization complications cost, routine monitoring, cost of CardioMEMS implantation, device and management | N/A | N/A |
| Sandhu et al, 2016 [52] | Societal | HF hospitalization cost, outpatient, catheterization and angiography, cost of CardioMEMS implantation, device and management | N/A | Not reported |
| Martinson et al, 2017 [53] | Payer | HF and non-HF hospitalizations, outpatients, cost of CardioMEMS implantation, device and management | N/A | N/A |
| Niewada et al, 2021 [54] | Public payer (the Polish National Health Fund (NHF)) and service provider | Cost of telerehabilitation | N/A | N/A |
| Calo et al, 2013 [55] | Hospital and patients | Costs of medical and nursing staff; depreciation costs of hospital machinery, instruments, and equipment used during in-hospital visits; cost increase due to RM service | Transportation and travel costs | Cost of lost employment income for the time spent for on in-hospital visits |
| Zanaboni et al, 2013 [56] | The health care system and societal. | Costs included urgent and nonurgent in-office visits, scheduled and unscheduled remote follow-ups, ER visits, hospitalizations, and diagnostic examinations | Transportation costs | Room and board, and wages lost by patients and family caregivers. |
| Jiang et al, 2021 [47] | US health care providers | Cost of hospitalization, outpatient costs, HTM costs | N/A | N/A |
| Boodoo et al, 2020 [48] | Public healthcare payer | ER costs, GP visit costs, drug costs, outpatient costs, hospitalization cost per admission, LOS days, Medly fixed cost for site implementation, Medly operational cost, Medly full kit cost, Medly bring-your-own-phone cost, Medly bring-your-own-everything cost | N/A | N/A |
| Cano Martin et al, 2014 [49] | The Ministry of Health of Spain | Cost of tool, cost of consultation in primary care, emergencies telephone consultation medication, home visits, diagnostic procedures and hospitalization | Transportation cost, management overhead | N/A |
| Hwang et al, 2018 [57] | Healthcare provider | Health care resource usage and program costs, labour costs, equipment costs, travel costs associated with the home visits, costs of acute day and overnight hospitalisation (ER visits, hospital readmissions and day procedures) | N/A | N/A |

N/A: not applicable; CFU: Conventional Follow-Up; DA: Diagnostic Algorithms; DAOH: Day Alive and Out of Hospital; DMP: Disease Management Programs; ER; Emergency Room; GDH: Geriatric Day Hospital; GP: General Practitioner; HF: Heart Failure; HFN; Heart Failure Nurse; HL: Health Lines intervention; HLM: Health Lines intervention plus in-house monitoring; HM: Home Monitoring; TM: Home Telemonitoring; LOS: Length of Stay; NHS: National Health System; NTS: Nurse Telephone Support; PCP: Primary Care Physician; RM: Remote Monitoring; RPM: Remote Patients Monitoring; SoC: Standard of Care; SCAD: Suivi Clinique A Domicile; TIM-HF2: Telemedical Intervention Management in Heart Failure II trial; TM: Telemonitoring; vs: versus; HR: Hazard Ratio

Sensitivity Analysis Summary

| **Author, Year** | **Study Design** | **Sensitivity Analysis** | **Most Influential Parameter in Sensitivity Analysis** |
| --- | --- | --- | --- |
| Caillon et al, 2022 [31] | CUA | DSA and PSA | HF Management cost |
| Thokala et al, 2013 [32] | CUA | PSA | Estimation of the effectiveness of HTM |
| Albuquerque de Almeida et al, 2022 [33] | CUA | DSA | Discount rate and utility due to hospitalization |
| Jiang et al, 2020 [34] | CUA | DSA | Probability of HF-related hospitalization in multidisciplinary care and all-cause mortality |
| Thokala et al, 2020 [35] | CUA | PSA | - |
| Voller et al, 2022 [36] | CEA | PSA | - |
| Boyne et al, 2013 [37] | CUA | PSA | - |
| Sydow et al, 2021 [38] | CUA | PSA | Total healthcare cost and effect on DAOH |
| Vestergaard et al, 2020 [39] | CUA | DSA | Adjusted complete case analysis |
| Grustam et al, 2018 [40] | CUA | PSA | - |
| Ruschel et al, 2018 [41] | CEA | PSA | - |
| Cui et al, 2013 [42] | CUA | DSA | Cost of intervention and hospitalization, and effectiveness of clinical outcomes |
| Hebert et al, 2008 [43] | CUA | PSA | - |
| Smith et al, 2008 [44] | CUA | DSA | Patient-specific mean imputation of missing data |
| Bocchi et al, 2018 [45] | CUA | DSA | Cost of intervention |
| Gonzalez-Guerrero et al, 2018 [46] | CUA | DSA | Transportation costs of the patient and caregiver costs in societal perspective |
| Cowie et al, 2017 [50] | CUA | DSA and PSA | HR reduction in mortality and cost of intervention |
| Schmier et al, 2016 [51] | CUA | DSA | Hospitalization and mortality rate |
| Sandhu et al, 2016 [52] | CUA | DSA and PSA | Device effectiveness and cost of intervention |
| Martinson et al, 2017 [53] | CUA | DSA and PSA | HF hospitalization and mortality rate |
| Niewada et al, 2021 [54] | CUA | PSA | Utility value and NYHA class |
| Calo et al, 2013 [55] | CMA | Not declared | None |
| Zanaboni et al, 2013 [56] | CUA | Not declared | None |
| Jiang et al, 2021 [47] | CUA | DSA | Outpatient cost for NYHA class I (Universal HTM vs Universal SoC and HTM for class II-IV vs universal SoC), HR of all-cause mortality for HTM vs SoC (HTM for class III-IV vs universal SoC) |
| Boodoo et al, 2020 [48] | CUA | DSA and PSA | The relative risk for mortality |
| Cano Martin et al, 2014 [49] | CUA | DSA | Effectiveness of intervention |
| Hwang et al, 2018 [57] | CUA | DSA and PSA | All-cause health care costs (including actual program costs, and aggregated costs of all-cause emergency visits, hospital readmissions and day procedures) |

SoC, defined by the European Society of Cardiology (ESC) and American Heart Association/ American College of Cardiology/ Heart Failure Society of America (AHA/ACC/HFSA), is the standard multidisciplinary management program, which includes regular planned follow-up for the purpose of safety and optimal drug dosing (standard care with or without drug or exercise prescription), early detection of decompensation and impact on disease progression that requires modification of the intervention or treatment regimen [28,29].

-: not stated; HF: heart failure; HR: heart rate; DSA: deterministic sensitivity analyses; PSA: probabilistic sensitivity analyses; N/A: Not applicable; CEA: cost-effectiveness analysis; CUA: cost-utility analysis; CMA: cost-minimization analysis; DAOH: Day Alive and Out of Hospital; HLM: health lines intervention plus in-house monitoring; NYHA: The New York Heart Association
